# Early surgical revascularization after acute myocardial infarction

**DOI:** 10.1186/1749-8090-8-S1-O173

**Published:** 2013-09-11

**Authors:** S Borovic, P Dabic, I Nesic, A Milutinovic, S Dzelebdzic, B Djukanovic

**Affiliations:** 1 Cardiac Surgery, Dedinje Cardiovascular Institute, Belgrade, Serbia; 2 School of Medicine, Belgrade, Serbia

## Background

Treatment of myocardial infarction has undergone great evolution since introduction of percutaneous coronary intervention. The purpose was to assess the outcome of patients with myocardial infarction undergoing early surgical revascularization with coronary artery bypass grafting (CABG).

## Methods

A total of 62 consecutive patients underwent CABG therapy within 14 days after the onset of myocardial infarction between September 2009 and January 2013 at our institution. Prospectively recorded preoperative, intraoperative, and postoperative data were retrospectively screened for in-hospital mortality and major adverse postoperative events (low cardiac output syndrome, prolonged mechanical ventilation, prolonged intensive care stay, hospital stay >7 days).

## Results

Overall in-hospital mortality was 3.2%. Low cardiac output syndrome was found in 27.4%, prolonged mechanical ventilation in 9,7%, prolonged intensive care stay in 48,4% and hospital stay >7 days in 64,5%. Age, female sex, EuroSCORE, extent of preoperative myocardial necrosis, low left ventricular ejection fraction and cardiogenic shock were the most potent predictors of major adverse postoperative events.

## Conclusions

CABG within 14 days after the onset of myocardial infarction can be performed with acceptable risk by incorporating adequate management strategies. However, age, female sex, extent of preoperative necrosis, preoperative cardiogenic shock and calculated high operative risk are major variables of morbidity results.

